# Geographically Localized Medicine House-Staff Teams and Patient Satisfaction

**DOI:** 10.1177/2374373518771361

**Published:** 2018-04-30

**Authors:** Zishan Siddiqui, Amanda Bertram, Stephen Berry, Timothy Niessen, Lisa Allen, Nowella Durkin, Leonard Feldman, Carrie Herzke, Rehan Qayyum, Peter Pronovost, Daniel J Brotman

**Affiliations:** 1 Division of General Internal Medicine, Johns Hopkins Medicine, Baltimore, MD, USA; 2 Division of Infectious Diseases, Johns Hopkins Medicine, Baltimore, MD, USA; 3 Armstrong Institute for Patient Safety and Quality, Johns Hopkins Medicine, Baltimore, MD, USA; 4 Division of Hospital Medicine, Virginia Commonwealth University, Richmond, VA, USA

**Keywords:** clinician–patient relationship, HCAHPS, interprofessional communication, patient satisfaction

## Abstract

**Background:**

Geographically localized care teams may demonstrate improved communication between team members and patients, potentially enhancing coordination of care. However, the impact of geographically localized team on patient experience scores is not well understood.

**Objective:**

To compare experience scores of patients on resident teams home clinical units with patients assigned to them off of their home units over a 10-year period.

**Participants:**

Patients admitted to any of the 4 chief resident staffed internal medicine inpatient service were included. Patients admitted to the house-staff teams’ home clinical unit comprised the exposure group and their patients off of their home units comprised the control patients.

**Measurement:**

Top-box experience scores calculated from the physician Hospital Consumer Assessment of Healthcare and Provider Systems (HCAHPS) and Press Ganey patient satisfaction surveys.

**Results:**

There were 3012 patients included in the study. There were no significant differences in experience scores with physician communication, nursing communication, pain, or discharge planning between the 2 groups. Patients did not report satisfaction more often with the time physicians spent with them on localized teams (48.6% vs 47.5%; *P* = .54) or that staff were better at working together (63.2% vs 61.3%; *P* = .29). This did not change during a 45-month period when the proportion of patients on home units exceeded 75% and multidisciplinary rounds were started.

**Conclusion:**

Patients cared for by geographically localized teams did not have better patient experience. Other factors such as physician communication skills or limited time spent in direct care may overshadow the impact of having localized teams. Further research is needed to better understand organizational, team, and individual factors impacting patient experience.

## Background

In most hospitals, care team members are distributed, infrequently in the same place at the same time, since physicians provide care to patients across multiple units while nurses are often unit based (1). This diffusion of physician geography is related in part to operational demands for moving patients efficiently from the emergency department to any appropriate open inpatient beds, irrespective of clinical unit. The resulting team dispersion is considered a barrier to improving teamwork (1). Its impact on patients’ perception of teamwork, communication, and care is not clear.

Strong team culture and communication between multidisciplinary teams are crucial to delivery of safe, effective, and high-quality care. Communication failure may be the root cause of two-third of sentinel events (2). Clinical teams that receive teamwork training and operationalize its principles show improved perception of teamwork, improved job satisfaction, and work environment (3). The Joint Commission requires evidence of physician–nurse collaboration for accreditation (4). Accreditation Council for Graduate Medical Education also recommends enhancing teamwork (5).

Physicians and nurses can improve collaboration by knowing whom to talk to, finding the right person, and coming together, which can be better accomplished when team members are in greater proximity (4). Interventions such as geographic localization of physicians, along with a daily goals of care checklist, interdisciplinary rounds, and team training have shown some success in improving teamwork and communication (6). Geographic localization of physician teams has shown to decrease the number of pages to residents, increased the ability by the team members to identify the correct physician or nurse, increased physician–nurse in-person communication, increased promptness of provider response, and better mutual understanding of plan of care and planned discharge date (7). The impact is felt to be mediated through proximity to patients and between members of the health-care team as well as through increased communication, decreased wasted time, and increased teamwork (8).

There is limited understanding of how geographic localization and related improvement in nurse–physician collaboration impacts patients’ perception of care. Using the Picker Patient Experience survey, studies have shown that patients on geographically localized team report better knowledge of diagnosis, felt that physicians better addressed their anxieties, and spent more time with them (9). However, it is unclear how the efforts related to geographic localization of physician teams improve delivery of patient-centered care by physicians and nurses as measured by the ubiquitous and publicly reported Hospital Consumer Assessment of Healthcare Provider and Systems (HCAHPS) survey. For example, the health systems investment in improving communication skills may overshadow the impact of localized teams.

Since it has been shown that improved resident physician communication with patients can result in significant impact on HCAHPS patient experience scores, we sought to study the impact of geographically localized resident physician teams on patient experience (10). We hypothesized that patients cared for by residents localized to their home clinical units, where these residents have their offices and spent significant part of their daily time/indirect care time, (11) would have significantly higher HCAHPS experience scores on physician, nursing, pain control, and discharge planning domains, when compared to patients localized to nonhome units for the residents. We also hypothesized that the relationship would be strengthened when strategies were deployed to achieve improved team collaboration and might be impacted by the degree of localization of patients to the home units.

## Methods

This was a retrospective analysis of prospectively collected HCAHPS and Press Ganey patient experience data for a single academic tertiary care hospital. The research project was reviewed and approved by the institutional review board (IRB).

### Participants

Hospitalized patients cared for by resident physicians in the department of medicine were included in the study. Specifically, patients who were admitted to the hospital on 1 of the 4 teaching inpatient services (called “firms”) and responded to HCAHPS patient experience survey were included in the study.

From January 2006 to June 2011, firms included 4 interns, 2 senior residents, and an assistant chief of service serving as the attending physicians. To comply with 2011 work hour regulations, the number of interns for each team increased to 5 between July 2011 and June 2014, without any changes in other team members. The team size decreased to 4 interns between July 2015 and March 2016. House staff are assigned to 1 of the 4 firms upon entry into the residency program and almost always stay with the firm through their 3 years of internal medicine residency training. A majority of the general medical inpatient rotations of the first- and third-year residents are on their firm’s inpatient service. Each firm has a designated office located on their home clinical unit. The offices include sufficient seating space, telephone sets, and computer terminals necessary for the inpatient team. The offices are used for teaching, team meetings, charting, communication, and care coordination.

### Exposure

Patients admitted to the firms and localized to the home clinical unit were considered the exposure group, since this group may benefit from physician proximity to patients and nurses. This group was labeled “geographically localized patients”. Patients admitted to a firm inpatient service on a clinical unit other than their designated home unit were considered the control group and were expected to receive care that is typical for patients on standard house-staff service. These patients were labeled “nongeographically localized patients”. The patients were distributed to various clinical units in a nonrandom fashion by the bed manager. For the sake of continuity, patients previously cared for by an inpatient firm service or belonging to one of house-staff outpatient panel were prioritized to be admitted to the firm’s inpatient service. Efforts were made to assign patients to the home clinical unit, but patients were assigned nongeographically to meet the operational needs to efficiently move the patients from the emergency department and to ensure appropriate distribution of patients to admitting interns. The pathway for triaging the patients to different inpatient teams and clinical units changed over the study as did the percentage of patients who were geographically localized (see below).

### Time Periods

We included patients admitted to the firms from January 2006, through March 2016. Additionally, based on some important structural changes in the firms, additional time points were defined: During a period from July 2012 to March 2016, a greater geographic localization of patients was achieved by making changes in the triage pathways to prioritize geographic localization. Additionally, mandatory multidisciplinary rounds were started during this time period on the home clinical unit, which likely further improved team collaboration. We called this period “increased localization period.” The differences between the exposure and the control group were analyzed separately for this period.

### Patient Experience Survey Instruments

Press Ganey patient and HCAHPS patient experience surveys were sent via mail in the same envelope starting in 2006. Fifty percent of the discharged patients were randomized to receive the surveys. The Press Ganey survey contained 33 items covering across several subdomains including room, meal, nursing, physician, ancillary staff, visitor, discharge, and overall experience. The HCAHPS survey contained 29 Centers for Medicare and Medicaid Services (CMS)-mandated items of which 21 are related to patient experience. The items related to nursing, physicians, pain control, discharge preparedness, and overall rating were utilized for the analysis. Three discharge related additional items were added to the HCAHPS survey over the years. The development and testing and methods for administration and reporting of the HCAHPS survey have been described previously (12). Press Ganey patient experience survey results have been reported in the literature (13).

### Outcome Variables

Press Ganey and HCAHPS patient experience survey responses on items related to nursing, physicians, pain control, discharge preparedness, and overall rating were the primary outcome variables of the study.

### Covariates

Age, sex, length of stay (LOS), and all-payer refined diagnosis-related group severity of illness were included as covariates.

### Statistical Analysis

“Percent top-box” scores were calculated for each survey item as the percentage of patients who responded”very good” for a given item on Press Ganey survey items and “always” or “definitely yes” or “9” or”10” on HCAHPS survey items. Centers for Medicare and Medicaid Services utilizes “percent top-box scores” to calculate payments under the value-based purchasing program and to report the results publicly. Numerous studies have also reported percent top-box scores for HCAHPS survey results (13).

To test our hypothesis, the percent top-box scores were calculated for geographically localized patients and nongeographic localized patients for the 2 study periods. *P* values for difference in top-box scores between geographic localized patients and nongeographic localized patients’percentage top-box scores, adjusted for age, sex, LOS, complexity of illness, and insurance type, were determined using logistic regression. All statistical analysis was performed using SAS Institute Inc’s (Cary, North Carolina) JMP Pro 11.0.0.

## Results

Among the HCAHPS survey respondents on the firm inpatient services, there were 1694 geographically localized patients and 1318 nongeographically localized patients over the 10-year study period. The response rates for geographically localized patients and nongeographically localized patients were similar (16.3% vs 16.1%; *P* = .72). There was no difference between the groups with regard to age, gender, race, severity of illness, self-reported health status, and education status. The 2 groups had similar length of stay as well (4.2 vs 4.1; *P* = 0.58; Table 1). During the period of increased efforts for geographic localization along with initiation of multidisciplinary rounds in July 2012, the proportion of geographic localized patients increased from 56.2% (for the entire study period) to 76.5%.

**Table 1.** Patient Characteristics.

Patient Characteristic	Geographically Localized Patients, n = 1694	Nongeographically Localized Patients, n = 1318	*P* Value
Mean age	62.3	61.6	.61
Female	58.3%	57.0%	.55
Nonwhite	64.9%	64.3%	.94
Length of stay	4.2	4.1	.58
APR-DRG SOI	2.6	2.6	.12
Self-reported health status			
** **Excellent or very good	24.2%	22.3	.81
Good	27.3%	29.1	
Fair	35.8%	36.1	
Poor	12.8%	12.5	
Education			
** **Less than high school	36.5%	31.0%	.10
** **High school graduates or some college	45.1%	50.6%	
** **4 or more year of college	18.4%	18.4%	

Abbreviation: APR-DRG SOI, All payer-refined diagnosis-related group severity of illness.

There was no difference in the physician domain scores, including those related to physician communication on HCAHPS and Press Ganey patient surveys between geographic localized patients and nongeographic localized patients for the entire study period and the increased localization period. Specifically, geographically localized patients did not report satisfaction more often with the time physicians spent with them (48.6% vs 47.5%; *P* = .54). This did not change with achievement of >76% localization of patients. During the same period (July 2012-March 2016), despite initiation of multidisciplinary rounds on physician home units, geographically localized patients did not feel that the staff worked better together (65.1% vs 65.6%; *P* = .86).

Pain-related experience, which may be impacted by a nurses ability to communicate patients pain rating to the physician and their ability to respond back, was again no different including during the increased localization period (68.9% vs 69.6%; *P* = .84). Similar to experience with physician communication, there were no differences between the groups with regard to nursing communication. Experience with discharge preparedness and overall hospital rating was also not different between the geographic localized patients and the nongeographic localized patients (Tables 2 and 3).

**Table 2.** Press-Ganey Satisfaction Scores for Geographically Localized Patients Versus Nongeographically Localized Patients.^a,b^

Satisfaction Domains	%Top Box for Entire Period^c^		*P* Value	%Top Box With Increased Localization^c^		*P* Value
	Geographically Localized Patients, n = 1694	Off-Unit Patients, n = 1318		Geographically Localized Patients, n= 827	Off-Unit Patients, n = 254	
Overall						
Staff worked together care for you	63.2	61.3	.29	65.1	65.6	.86
Likelihood recommending hospital	64.8	62.1	.12	67.9	65.4	.46
Overall rating of care given	67.0	64.2	.15	70.3	67.3	.37
Physician						
Time physician spent with you	48.6	47.5	.54	52.5	50.6	.60
Physician concern questions/worries	55.6	54.5	.43	59.6	57.4	.54
Physician kept you informed	55.0	52.5	.18	58.4	54.6	.29
Friendliness/courtesy of physician	62.9	60.8	.25	66.7	62.2	.18
Skill of physician	65.0	62.9	.25	68.9	66.5	.49
Pain control						
How well your pain was controlled	48.6	47.3	.48	49.7	44.6	.18
Nursing						
Friendliness/courtesy of the nurses	67.8	68.5	.66	71.1	73.5	.44
Promptness response to call	51.0	50.9	.95	52.7	53.7	.77
Nurses’ attitude toward requests	59.2	60.1	.62	61.6	62.3	.72
Attention to special/personal needs	56.6	56.5	.94	59.1	58.2	.78
Nurses kept you informed	56.0	55.8	.91	59.2	60.8	.65
Skill of the nurses						
Discharge						
Extent felt ready discharge	49.3	46.7	.16	53.1	45.7	.04
Speed of discharge process	36.2	39.6	.12	38.2	36.7	.70
Instructions care at home	54.7	54.6	.96	57.3	54.6	.44

^a^Entire study period is January 2006 and March 2016.

^b^Increased localization occurred between July 2012 and March 2016.

^c^% Top Box = Percentage of patients who gave response at the highest level for the survey item.

**Table 3.** HCAHPS Satisfaction Scores for Geographically Localized Patients Versus Nongeographically Localized Patients.

Satisfaction Domains	%Top Box^a^ Entire Period^b^		*P* Value	%Top Box With Increased Localization^c^		*P* Value
	Geographically Localized Patients, n = 1694	Off-Unit Patients, n = 1318		Geographically Localized Patients, n = 827	Off-Unit Patients, n = 254	
Nursing communication						
Nurses treated with courtesy/respect	81.8	80.6	0.39	83.3	85.8	.33
Nurses listened	71.9	71.9	0.99	73.7	77.1	.25
Nurses explained	70.8	70.9	0.97	72.2	75.7	.27
Physician communication						
Doctors treated with courtesy/respect	82.1	83.1	0.46	83.2	83.6	.85
Doctors listened	74.3	74.7	0.80	75.4	78.0	.38
Doctors explained	71.2	70.7	0.75	70.9	72.3	.64
Miscellaneous						
Pain well controlled	52.3	55.3	0.18	54.7	47.4	.07
Staff do everything help with pain	66.7	70.8	0.04	68.9	69.6	.84
Staff talk about help when you left	79.5	74.4	0.002	81.4	81.5	.95
Info re symptoms to look for	87.8	86.2	0.20	90.0	88.3	.45
Overall						
Rate hospital (0-10)	67.7	66.9	0.65	70.0	69.9	.96
Recommend hospital	71.3	68.5	0.09	72.6	71.2	.63

Abbreviation: HCAHPS, Hospital Consumer Assessment of Healthcare Provider and Systems.

^a^% Top Box = Percentage of patients that gave response at the highest level for the survey item (For “Rate hospital” responses 9 and 10 were considered top box).

^b^Entire study period is January 2006 and March 2016.

^c^Increased localization occurred between July 2012 and March 2016.

## Discussion

We found no difference in experience between geographically localized patients on internal medicine residents home units when compared to patients off of their home units. Contrary to our hypothesis, we found no improvement in patient–physician communication, patient–nurse communication, patients’ discharge preparedness, or their experience with pain control. The differences remained insignificant even when the rate of geographically localized patients increased to 75.6%, and multidisciplinary rounds were introduced on the home units, factors that decreased team dispersion and were intended to improve interdisciplinary collaboration.

Our results appear inconsistent with the findings of some of the earlier studies. Designs of these previous studies use either a “pre–post” design in which experience data from patients cared for on newly created localized unit were compared to archival data of unit(s) that were not localized or cross-sectional designs compared scores from localized unit(s) to other units active at the same time (9,14). In both of these designs, the doctors caring for the patients on each unit were different. Our study design is unique in that we compared patients cared for by the same resident team on their home unit (geographic localized patients) with patients cared for by them on other units (nongeographic localized patients).

In another study evaluating patient experience, Olson et al (9) took advantage of a natural experiment that required consolidation of house-staff patients to a single clinical unit because of 2011 resident duty hour restrictions. In this pre–post analysis, they report improved patient experience with physicians as one of their outcomes. However, this was a 4-month study with only 153 patients combined in the 2 arms. The authors note significant variability in diagnosis. Significant physician variability likely occurred during this short study period, and both these factors could have impacted patient experience. Of note, they included items from both the HCAHPS survey and the Picker Patient Experience survey and found no statistical differences in patient ratings for physician, nurses, and overall care on HCAHPS items. This is consistent with our findings.

Resident teams in our study did not achieve full localization of patient care. There was a significant improvement to 75.6% during the increased localization period. Fanucchi et al (7) suggest a dose-dependent relationship with localization such that full geographic localization achieved the greatest improvement in physician–nursing communication, but the partially localized team fared better than standard teams. However, we did not find this to be the case for patient–physician or patient–nursing communication.

Although resident training in communication along with regular feedback and recognition has shown to improve HCAHPS scores, (10) it appears that increased proximity to patients and nursing team does not produce similar results. Residents have been noted to spend an average of 4.3 minutes per patient at some clinical sites. They spend only 12.3% of their time in direct patient care and only 0.6% of their time on patient education and family meetings (11). It would not be surprising that additional efficiencies generated by geographic localization are devoted to increasingly complex care coordination activities and indirect patient care with minimal change in the quantity or quality of direct patient care. Patients in our study also did not perceive increased face-to-face time with physicians on geographic units. The impact of inpatient resident team may have been further diluted by patients’ inclusion of performance by the consultant teams in their physician evaluations. In addition, the impact of proximity on patient experience may be a weaker effect than the organizations culture and practices for physician communication.

Our study has some limitations. This was only a single-center study. Furthermore, inpatient team structure, with 4 interns, 2 senior residents, and 1 attending is an uncommon one. As a result, the findings may not be generalizable to other hospitals. However, we are unaware of any mechanism by which the team structure might have impacted our key findings. Our study is limited by lack of randomization. Also, the surveys are sent approximately 1 week after discharge and often filled much later. Some of the underlying differences may not be reported because of lack of recall. However, the patient experience surveys are routinely administered and provide a relatively large, readily available data set to conduct an adequately powered analysis. Hospital Consumer Assessment of Healthcare Provider and Systems surveys used in our study have low response rates. However, this is the case for HCHAPS surveys in general across the nation, and these results are deemed important and valid enough to justify CMS pay-for-performance incentives.

Although our study did not show any adverse consequences on HCAHPS scores as a result of nongeographic localization of patients, previously demonstrated benefits of geographic localized teams with regard to collaboration and communication may be a strong argument in favor of wider adoption. Hospitals should continue to focus on improving physician etiquette and communication skills to improve patient-centered care and HCAHPS scores. Additional interventions that take advantage of geographic localization of patients to enhance patient–physician communication, such as bedside patient handoff and evening patient rounds, may enhance patient-centered care.
